# Letter of Orfila to the Director of the School of Pharmacy of Paris

**Published:** 1853-07

**Authors:** 

**Affiliations:** Paris


From the American Journal of Pharmacy.

*Letter of Orfila to the Director of the School of Pharmacy of Paris.*

PARIS, Jan. 1st, 1853.

MR. DIRECTOR AND DEAR COLLEAGUE:—Thirty-two years as an examiner in the Special School of Pharmacy of Paris, has enabled me to appreciate the distinguished merit and the honorable zeal of its professors, as well as the remarkable aptitude of most of the candidates who have assiduously followed their instruction. I will preserve, while I live, a grateful remembrance of the happy relations which have unceasingly existed between you, your colleagues, and myself, and I am happy in feeling able at this time to furnish you a proof of the desire which animates me, by contributing some little to enhance the renown of an institution which is honorable to France, and of which you are the worthy director.

I place at your disposal an inscription of 500 francs per annum in the three per cents, designed to found a prize of 1000 francs, which shall be awarded every two years, dating from the commencement of the session for the year 1856.—This inscription represents a sum of 14,000 francs, (at 84 the cost price.)

The prize is not to be divided. If it is not awarded, the same question is to be again put up for competition, and the prize will be then 2000 francs. If the prize is not gained the second time, the same question will be proposed for the third time, the prize being 3000 francs; if, notwithstanding the delays, the question has not been properly answered, and the prize not adjudged, the sum of 3000 francs is to be paid in to the treasury of the Association of Physicians of the department of the Seine, which I founded in 1853.

Allow me, Mr. Director, to state briefly a certain number of questions, which, in my opinion, should be first proposed.

**1st.** To extract from the most important compound medicines all the proximate principles, or other active substances belonging to them. We should not believe, because an alcaloid, or any other body possessing a degree of power, has been obtained from a drug, that science can do no more; in fact, a substance extracted from a medicine, may account for a certain portion of its therapeutic effects, but often many other effects are due to ingredients not isolated. It is necessary to be quite certain in this regard, that all which relates to the action of compound medicines on the same animal economy may be completely understood, and the part which the different active principles take in this action ascertained. This question will furnish, without doubt, a number of subjects for prizes.

2d. To determine, by experiment, what are the substances in the several kingdoms which should never be associated in the same formulæ, because they decompose each other, with products completely inert. To ascertain, on the contrary, which are the substances, which in combining and in decomposing, yield medicines gifted with a certain activity useful to the physician. To indicate the kind of changes that occur in these various substances, and the nature of the new compounds formed.

3d. To explain the best process for discovering certain sophistications which have not yet been the object of serious study.

4th. To decide what modifications certain drugs, of vegetable and animal origin, will undergo by continued exposure to heat and light, and to dry or moist air, etc., and to say whether the products which result from the alteration of these drugs will cause accidents, in case they are employed in medical practice.

5th. To analyze the saliva, the urine, and perspiration in the principle acute diseases called specific, so as to be able to state the changes that occur in those fluids; added to which, the expired air should be examined.

6th. To search if, in lying-in women, the milk partially abandons the galactiferous organs, and is carried to other vessels, and especially if, in the so called milk diseases, to which women recently confined are sometimes subject, the milk has been carried to the bladder, into certain serous cavities, &c.

7th. To submit the mineral waters, yet but little known to analysis, and to restudy those which enjoy great celebrity, with the object of ascertaining whether some new active substances cannot be discovered. If the problem proposed by the Academy of Medicine, in 1851, on the occasion of the Capuron prize, has not received a satifactory solution, it may be removed from competition.

Such, Mr. Director, are the questions which need elucidation. The demands of science will lead you, I have no doubt, to propose other questions, either before or after those which have been indicated. I am satisfied of the sagacity of the Professors in this matter, and accept in advance the programme they may offer.

Receive, my dear colleague, the assurance of my distinguished consideration and affectionate regard. ORFILA.

